# Adult T-cell leukemia/lymphoma: molecular pathogenesis, emerging therapies, and future directions

**DOI:** 10.3389/fonc.2026.1794939

**Published:** 2026-03-31

**Authors:** Youssef Samaha, Hannah Hugo, Roshan Asrani

**Affiliations:** 1 Department of Medicine, Icahn School of Medicine at Mount Sinai, NYC Health +Hospitals/Elmhurst, New York, NY, United States; 2 Department of Hematology/Oncology, Icahn School of Medicine at Mount Sinai, New York, NY, United States

**Keywords:** adult T-cell leukemia/lymphoma (ATLL), antiviral therapy, CCR4, HTLV-1, immunotherapy, mogamulizumab, NF-κB

## Abstract

Adult T-cell leukemia/lymphoma (ATLL) is a rare and aggressive malignancy of mature T-cells caused by chronic infection with Human T-cell Leukemia Virus Type 1 (HTLV-1). Despite advances in understanding its pathobiology, ATLL remains associated with poor clinical outcomes, largely due to intrinsic chemoresistance and immune evasion mechanisms driven by viral oncogenes and acquired genetic alterations. The discovery of frequent mutations in TCR/NF-κB, JAK/STAT, and apoptotic pathways has unveiled novel therapeutic vulnerabilities, while agents such as mogamulizumab have demonstrated clinical efficacy in relapsed settings. Antiviral therapies and allogeneic hematopoietic stem cell transplantation (allo-HSCT) represent pillars of treatment for selected patients, but durable remissions remain rare. This review comprehensively summarizes the molecular underpinnings of ATLL, highlights the evolving therapeutic landscape, and discusses emerging research directions aimed at improving patient outcomes.

## 1 Introduction

Adult T-cell leukemia/lymphoma (ATLL) is an aggressive peripheral T-cell neoplasm etiologically linked to chronic infection with Human T-cell Leukemia Virus Type 1 (HTLV-1), first molecularly characterized following viral isolation in 1982 (1). First clinically described in Japan in the 1970s, ATLL represents a well-established model of human retroviral oncogenesis, supported by molecular and integration site analyses demonstrating clonal expansion of HTLV-1–infected T cells (1, 2). Global HTLV-1 infection is estimated to affect several million individuals, with marked geographic clustering in southwestern Japan, the Caribbean basin, parts of South America, and sub-Saharan Africa (3). Longitudinal studies demonstrate that elevated proviral load is associated with increased risk of ATLL development, although only a minority of carriers progress to malignancy (4, 5).

Clinically, ATLL encompasses heterogeneous subtypes defined by the Shimoyama classification—acute, lymphoma, chronic, and smoldering—each associated with distinct clinical behavior and prognosis (6). Conventional chemotherapy regimens, such as CHOP, have demonstrated inferior outcomes compared with dose-intensified regimens in prospective trials, including JCOG9801, underscoring intrinsic chemoresistance in aggressive disease (7). Therapeutic strategies now include antiviral therapy (zidovudine/interferon-α), anti-CCR4 monoclonal antibody therapy, and allogeneic hematopoietic stem cell transplantation (allo-HSCT), each supported by prospective or registry-level data, yet long-term survival remains limited outside selected transplant-eligible populations (8–11).

At the molecular level, HTLV-1 encodes viral proteins such as Tax and HBZ, which exert complementary oncogenic functions: Tax promotes NF-κB activation and anti-apoptotic signaling, whereas HBZ sustains proliferation and modulates host transcriptional programs *in vivo* (12–14).

Integrated genomic analyses have identified recurrent somatic alterations in TCR/NF-κB pathway components, CCR4, epigenetic regulators, and immune surveillance genes, with clinicogenomic correlations demonstrating prognostic relevance of specific mutational patterns (15–17).

In this review, we synthesize primary mechanistic and clinicogenomic evidence to delineate how HTLV-1 viral programs intersect with recurrent, potentially targetable somatic lesions. We further stratify therapeutic evidence by trial design, geographic context, and patient selection, explicitly addressing generalizability beyond Japan-dominant datasets. Particular attention is given to treatment-specific risk trade-offs, including allo-HSCT–associated non-relapse mortality and reported hyperprogression following PD-1 pathway blockade (18). By integrating biologic insight with evidence-tiered clinical data, we aim to provide a critically appraised framework rather than a descriptive summary of existing literature.

### 1.1 Scope and definitions

In this review, we focus on HTLV-1–associated adult T-cell leukemia/lymphoma (ATLL/ATL) as a distinct mature T-cell neoplasm arising from chronic HTLV-1 infection. We organize clinical discussions using the Shimoyama subtype classification (acute, lymphoma, chronic, smoldering), as this framework remains central to clinical decision-making and is referenced throughout major treatment paradigms (6). Unless otherwise specified, “ATLL” refers to the malignant entity and not other HTLV-1–associated diseases (e.g., HTLV-1–associated myelopathy/tropical spastic paraparesis). For consistency with the literature, we use “ATLL” and “ATL” interchangeably when citing historical studies, while maintaining a unified subtype framework across sections.

## 2 Epidemiology and viral pathogenesis

### 2.1 Global epidemiology of HTLV-1 and ATLL

Human T-cell Leukemia Virus Type 1 (HTLV-1) infects several million individuals worldwide, with epidemiologic clustering in defined endemic regions (3). The prevalence of HTLV-1 is geographically heterogeneous, with endemic regions including southwestern Japan, the Caribbean basin, parts of Central and South America, sub-Saharan Africa, and localized areas in the Middle East and Australo-Melanesia (3). In these regions, the seroprevalence among adults may reach up to 5–10% (3).

The lifetime risk of developing ATLL among HTLV-1 carriers is estimated at approximately 2–5%, based on long-term observational cohorts in endemic regions (19). Risk is modulated by age at acquisition (higher with vertical transmission), host immune response, and proviral load levels, with prospective data demonstrating that elevated proviral load independently predicts disease progression (4, 5).

Vertical transmission through prolonged breastfeeding remains the predominant route of infection in endemic areas (20). Other transmission modes include sexual contact, blood transfusions, and parenteral exposure (3). Implementation of blood product screening and public health strategies modifying breastfeeding practices have reduced new infections in several endemic regions, particularly Japan (3).

### 2.2 Viral oncogenesis and mechanisms of transformation

HTLV-1 is a complex retrovirus that integrates its proviral genome into host DNA, establishing lifelong infection primarily within CD4+ T-cells (1, 2). Although infection alone is insufficient for malignant transformation, longitudinal and molecular studies demonstrate that persistent viral gene expression and clonal expansion over decades precede malignant evolution (2, 21).

Two viral proteins, Tax and HBZ (HTLV-1 bZIP factor), play central roles in ATLL pathogenesis. Tax is a viral transactivator that promotes T-cell proliferation through constitutive activation of the NF-κB signaling, stabilization of anti-apoptotic mediators such as MCL-1, and interference with tumor suppressor pathways (12, 22). Tax expression is often downregulated or intermittently expressed in established ATLL, with experimental evidence supporting immune selection pressure as a contributing mechanism (21, 23).

In contrast, HBZ is consistently expressed in ATLL and promotes proliferation and persistence of infected T cells, as demonstrated in *in vitro* and *in vivo* models, while modulating host transcriptional and epigenetic programs (13, 14). HBZ expression has been associated with regulatory T-cell–like phenotypes and altered immune signaling, although the precise contribution to immune checkpoint regulation remains incompletely defined (14).

HTLV-1 integration preferentially occurs in transcriptionally active regions of the genome, contributing to host gene dysregulation and clonal expansion (2). Clonal expansion of infected T cells under selective pressures precedes the acquisition of recurrent somatic mutations identified by integrated genomic analyses, linking viral persistence to genetically fixed malignant transformation (15–17).

## 3 Molecular pathogenesis and genomic landscape

### 3.1 Overview of genetic alterations in ATLL

Adult T-cell leukemia/lymphoma (ATLL) is distinguished by a complex interplay between viral oncogenesis and the accumulation of somatic genetic alterations defined by integrated genomic sequencing studies (15, 16). Advances in next-generation sequencing have elucidated the mutational spectrum of ATLL, revealing recurrent involvement of TCR/NF-κB signaling, JAK/STAT activation, immune evasion pathways, and epigenetic regulators (15–17).

Whole-exome sequencing (WES) studies have consistently identified high-frequency mutations in genes such as CCR4, PLCG1, PRKCB, CARD11, STAT3, TP53, and CDKN2A (15–17). Mutations typically involve gain-of-function alterations in signaling mediators (e.g., CCR4, PLCG1, PRKCB) and loss-of-function events in tumor suppressors (e.g., TP53, CDKN2A), contributing to clonal selection and disease progression (15, 17).

Importantly, the genomic profile of ATLL varies between different geographic regions and clinical subtypes, with clinicogenomic analyses demonstrating distinct mutational patterns and prognostic correlations across acute, lymphoma-type, and indolent forms (16, 17).

### 3.2 Key mutated pathways

#### 3.2.1 TCR/NF-κB signaling pathway

The T-cell receptor (TCR) signaling cascade is frequently deregulated in ATLL. Mutations in PLCG1, PRKCB, CARD11, and VAV1 lead to constitutive activation of the NF-κB pathway through proximal TCR signaling amplification and downstream IκB kinase activation (15, 16, 24). These alterations are considered central drivers of malignant transformation, as demonstrated by functional studies linking these mutations to enhanced NF-κB transcriptional activity and cytokine-independent growth (15, 24).

#### 3.2.2 JAK/STAT pathway

Aberrant JAK/STAT signaling has been implicated in ATLL through recurrent activating mutations in STAT3, STAT5B, JAK1, and JAK3 identified in large-scale sequencing cohorts (15–17). Activation of this pathway enhances cytokine-independent survival and contributes to immune escape. Notably, STAT3 mutations are associated with distinct transcriptional programs and adverse clinical outcomes in clinicogenomic analyses (16, 17).

#### 3.2.3 Cell cycle regulation

Mutations or deletions affecting TP53, CDKN2A, and RB1 impair normal cell cycle checkpoints and apoptosis, favoring unchecked proliferation (25). TP53 inactivation, in particular, is a negative prognostic marker with integrated genomic studies demonstrating inferior survival among TP53-mutated cases (17).

#### 3.2.4 Epigenetic dysregulation

Epigenetic alterations are emerging as significant contributors to ATLL pathogenesis. Mutations in TET2, EP300, CREBBP, and other chromatin modifiers have been identified in sequencing studies, alongside Polycomb-mediated epigenetic repression including loss of miR-31 leading to NIK-dependent NF-κB activation (15, 24, 26).

#### 3.2.5 Immune escape mechanisms

Mutations that promote immune evasion, including gain-of-function CCR4 mutations, PD-L1 structural variations, and disruption of antigen presentation pathways, enable ATLL cells to escape host immune surveillance (15, 16, 27). These immune-evasive alterations provide a mechanistic framework for the paradoxical hyperprogression observed following PD-1 blockade in ATLL (18).

### 3.3 Integration of viral and somatic events

The interplay between HTLV-1 viral proteins and host genetic alterations is central to ATLL biology. Early infection events driven by Tax and HBZ initiate T-cell proliferation, genomic instability, and survival signaling, thereby facilitating the accumulation of oncogenic somatic mutations over time (13, 15, 28). Over time, the cumulative burden of genetic lesions leads to full transformation and aggressive disease behavior (29).

### 3.4 Mutational landscape summary

**Figure 1:**
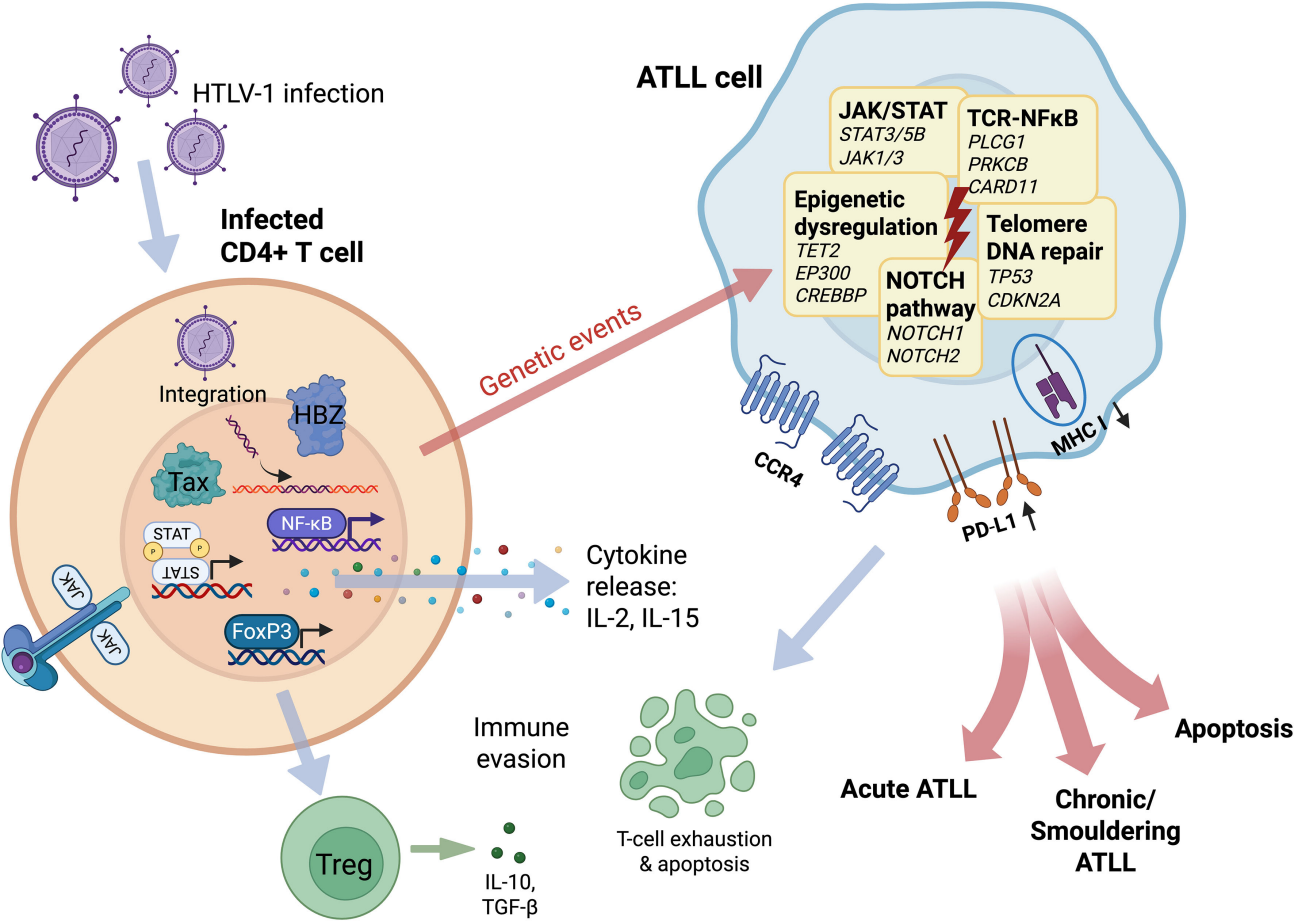
Pathogenesis of adult T-cell leukemia/lymphoma (ATL) from HTLV-1 infection. HTLV-1 infects CD4+ T cells, where viral oncoproteins Tax and HBZ drive malignant transformation. Tax activates NF-κB signaling and promotes cytokine release, while HBZ induces FoxP3 expression, conferring a Treg phenotype, facilitating immune evasion. Viral RNA reverse transcribes to DNA and integrates into cellular DNA driving clonal expansion. Accumulation of genetic events - including mutations in TCR-NFκB signaling pathway components, constitutive JAK/STAT activation, NOTCH pathway activation, epigenetic dysregulation and tumor suppressor inactivation - drives progression to overt ATLL. The resulting ATLL cells evade immune surveillance through CCR4 and PD-L1 overexpression, MHC-I downregulation, and induction of T-cell exhaustion. Depending on the balance between oncogenic signaling, immune pressure, and apoptotic responses, these processes culminate in acute, chronic/smoldering, or apoptotic ATLL phenotypes.

Recent integrated genomic analyses have clarified the mutational frequencies in ATLL. CCR4 mutations are the most frequent (~30–40%), as demonstrated in integrated genomic cohorts, followed by recurrent alterations in PLCG1, PRKCB, STAT3, and TP53 (15–17). The identification of these recurrent mutations provides not only mechanistic insights but also therapeutic opportunities, particularly in the context of precision medicine approaches, where mutational profiling increasingly informs prognostication and therapeutic targeting strategies (16, 17). **Figure 1** summarizes the progression from HTLV-1 infection to ATLL development, integrating key viral oncogenic mechanisms, recurrent somatic alterations, and downstream immune-evasion and apoptotic pathways.

## 4 Clinical presentation and diagnosis

### 4.1 Clinical subtypes

The clinical presentation of Adult T-cell leukemia/lymphoma (ATLL) is heterogeneous and has been historically classified according to the Shimoyama criteria into four major subtypes: acute, lymphomatous, chronic, and smoldering (6).

Acute ATLL: The most aggressive form, characterized by high tumor burden in peripheral blood, lymphadenopathy, hepatosplenomegaly, skin lesions, hypercalcemia, and opportunistic infections (6, 29). Central nervous system involvement may occur and portends a poor prognosis (30).

Lymphomatous ATLL: Presents predominantly with extranodal lymphadenopathy and minimal peripheral blood involvement. Hypercalcemia and skin lesions are less common compared to the acute form (6, 29).

Chronic ATLL: Features mild lymphocytosis, skin lesions, and lymphadenopathy without significant organ dysfunction. It is further subdivided into favorable and unfavorable types based on laboratory parameters such as serum lactate dehydrogenase (LDH) and calcium levels (6, 29).

Smoldering ATLL: The least aggressive form, often presenting with skin or pulmonary lesions without hypercalcemia, organomegaly, or marked hematologic abnormalities (6).

The distinction between subtypes is clinically significant, as it directly influences prognosis and therapeutic decision-making (29). The key clinical, laboratory, and prognostic features of the major ATLL subtypes are summarized in **Table 1**.

**Table 1:** Clinical subtypes of adult T-cell leukemia/lymphoma (ATLL): key features, laboratory findings, and prognostic implications.

Feature	Acute ATLL	Lymphomatous ATLL	Chronic ATLL – favorable	Chronic ATLL – unfavorable	Smoldering ATLL
Prevalence	~55%	~20%	~10–15%	~10%	~5%
WHO 2022 Classification Mapping	Aggressive ATLL (leukemic subtype)	Aggressive ATLL (lymphoma subtype)	Indolent ATLL	Indolent/Intermediate ATLL	Indolent ATLL
Peripheral Blood Involvement	Marked leukemic phase; many “flower cells”	Minimal or absent	Lymphocytosis; atypical cells	Higher lymphocyte counts with atypia	Minimal or absent
Lymphadenopathy	Common, generalized	Prominent and often bulky	Mild	Moderate	Rare or mild
Skin Involvement	Very common	Common	Frequent patches/plaques	Frequent, more infiltrative lesions	Hallmark feature—patches/plaques common
Organ Involvement	Liver, spleen, CNS, lung, GI	Nodal ± extranodal	Mild organomegaly	Organomegaly more frequent	Skin and lung only
Hypercalcemia	Very common (40–70%)	Occasional	Rare	More frequent	Absent
LDH Levels	High	High	Normal	Elevated	Normal
Opportunistic Infections	Very common	Moderate	Mild	Moderate	Rare
HTLV-1 Proviral Load	High	High	Intermediate	High	Low–intermediate
Immunophenotype	CD3+, CD4+, CD25+, CCR4+, FoxP3+; CD7 loss frequent	Same as acute	Same as acute	Same as acute; CD7 loss more frequent	Same as acute but lower burden
Genomic Features	High-risk mutations: TP53, CDKN2A, PLCG1, PRKCB, STAT3	Similar to acute	Low mutational burden	Higher frequency of TP53/CDKN2A	Lower-risk genomic alterations
Diagnostic Criteria	≥5% abnormal lymphocytes, HTLV-1+, TCR clonality	Tissue biopsy; HTLV-1+, TCR clonality	≥5% atypical lymphocytes; low LDH; normal calcium	Same as favorable + abnormal LDH or calcium	<5% atypical lymphocytes; skin/pulmonary disease
Transformation Risk	N/A	Already aggressive	Low (<10%)	Significant risk (30–40%)	Low progressing to 15–25% over years
Clinical Course	Rapid progression	Aggressive	Indolent	Intermediate, tendency to progress	Highly indolent
Median Overall Survival	6–10 months	10–13 months	~5 years	~2 years	>5–10 years
Optimal Therapy	Multi-agent chemo (e.g., mLSG15) → HSCT in CR1	Multi-agent chemo → HSCT	AZT + IFN, or observation	AZT + IFN ± chemo	Observation or antivirals
Response to AZT + IFN	Limited	Poor	Excellent	Intermediate	Good
Response to Chemotherapy	Poor without HSCT	Poor without HSCT	Not indicated	Sometimes needed	Not indicated

Subtype criteria based on Shimoyama et al. (6). Reported prevalence and outcome ranges are approximate and vary by cohort, geography, and treatment era.

Notably, emerging clinicogenomic data suggest that traditional Shimoyama clinical subtypes incompletely capture the biological heterogeneity of ATLL, as specific mutational patterns (e.g., TP53 alterations, CCR4 mutations, high-risk genomic clusters) refine risk beyond clinical phenotype alone (15–17). This evolving integration of molecular and clinical classification has important implications for future risk-adapted therapeutic algorithms.

### 4.2 Diagnostic approach

The diagnosis of ATLL requires the integration of morphologic, immunophenotypic, serologic, and molecular findings (5).

Morphology: Peripheral blood smears often demonstrate atypical lymphocytes with deeply indented or lobulated nuclei, known as “flower cells” (6). Bone marrow involvement is common in acute and chronic subtypes but may be variable in lymphomatous cases (6, 31).

Immunophenotyping: ATLL cells typically express a mature CD4+ T-cell phenotype with strong positivity for CD4, CD25 (IL-2 receptor α-chain), and CCR4 (27, 32). Other common markers include CD45RO and FoxP3, reflecting a regulatory T-cell–like phenotype (13, 33). Aberrant loss of T-cell antigens such as CD7 or CD3 may be observed (32).

Serologic Testing: Confirmation of HTLV-1 infection via serology (enzyme-linked immunosorbent assay, ELISA) followed by Western blot or PCR is essential for diagnosis (1, 4).

Molecular Studies: Molecular detection of monoclonal T-cell receptor (TCR) gene rearrangements and quantification of HTLV-1 proviral load can support the diagnosis and help distinguish ATLL from other mature T-cell neoplasms (4, 5). Advanced techniques such as next-generation sequencing (NGS) are increasingly employed for mutational profiling, prognostication, and clinical trial enrollment (15–17).

### 4.3 Differential diagnosis

The differential diagnosis of ATLL includes peripheral T-cell lymphoma, not otherwise specified (PTCL-NOS), Sézary syndrome, and other cutaneous T-cell lymphomas (6, 29). Chronic viral infections (e.g., Epstein-Barr Virus–associated T-cell proliferations) and reactive lymphocytosis due to infections or autoimmune disease may also mimic ATLL. Distinguishing ATLL from these entities relies heavily on demonstration of HTLV-1 infection and clonality assessment (4, 5).

## 5 Prognosis

### 5.1 Prognostic factors

Prognosis in Adult T-cell leukemia/lymphoma (ATLL) remains poor overall, with substantial heterogeneity driven by clinical subtype, disease burden, host factors, and underlying molecular architecture (6, 16, 29).

Clinical subtype is a major determinant of outcome. In the original Shimoyama classification and subsequent cohort analyses, acute and lymphomatous subtypes demonstrate significantly inferior survival compared with chronic and smoldering disease (6, 29). However, indolent subtypes are not biologically inert; longitudinal studies demonstrate that chronic ATLL, particularly unfavorable chronic disease, carries a meaningful risk of progression to aggressive forms (5, 29).

Performance status and laboratory markers are consistently incorporated into prognostic models. The ATL Prognostic Index (ATL-PI) identified poor performance status, elevated LDH, advanced age, and hypoalbuminemia as independent adverse variables (29), and modified indices in older populations have confirmed the prognostic contribution of similar parameters (34). Hypercalcemia and elevated LDH reflect tumor burden and aggressive biology and correlate with inferior survival across subtypes (29).

HTLV-1 proviral burden represents an important biological correlate of disease evolution. Prospective and retrospective analyses demonstrate that higher proviral load and clonal expansion metrics are associated with increased risk of ATLL development and progression from indolent states (4, 5), and more refined quantification approaches further support the biological relevance of integration burden (14).

Importantly, recent integrated genomic studies have demonstrated that recurrent somatic alterations carry independent prognostic implications. TP53 aberrations and disruptions in tumor suppressor pathways are associated with inferior outcomes (35, 36), while large-scale clinicogenomic analyses have linked mutational profiles—including alterations in TCR/NF-κB and immune-related pathways—to survival differences and treatment resistance patterns (15–17). These data suggest that genomic architecture may refine risk stratification beyond traditional clinical indices, although prospective validation outside Japanese cohorts remains limited.

### 5.2 Prognostic indices

Multiple prognostic models have been developed to stratify patients with ATLL using clinically accessible variables (29, 34). The principal clinically used prognostic models, including ATL-PI and JCOG-PI, are summarized in **Table 2**.

**Table 2:** Prognostic indices in adult T-cell leukemia/lymphoma (ATLL): ATL-PI and JCOG models.

Index	Components	Risk stratification	Definition of risk groups	Associated outcomes
ATL-PI (Adult T-Cell Leukemia Prognostic Index)	• Age • ECOG Performance Status • Serum Albumin • Serum LDH	Low-, Intermediate-, High-Risk	Low-Risk: ATL-PI ≤ 1 Intermediate-Risk: ATL-PI 1–2 High-Risk: ATL-PI ≥ 3	• Low-Risk: Median OS > 2–3 years • Intermediate-Risk: Median OS ~1–2 years • High-Risk: Median OS < 1 year
JCOG Prognostic Index (JCOG-PI)	• Hypercalcemia • ECOG Performance Status ≥ 2 • High LDH • Organ Involvement (liver, spleen, GI tract)	Low-, Intermediate-, High-Risk	Low-Risk: 0–1 adverse factor Intermediate-Risk: 2 adverse factors High-Risk: ≥3 adverse factors	• Low-Risk: Median OS ~2 years • Intermediate-Risk: Median OS ~1 year • High-Risk: Median OS < 0.5 years

ATL-PI derived from Katsuya et al. (29). Modified index in older populations: Fuji et al. (34).

The ATL-PI, derived from large Japanese cohorts, stratifies patients into low-, intermediate-, and high-risk categories with significantly distinct survival outcomes based on age, performance status, LDH, and albumin (29). Subsequent analyses have proposed modified indices applicable to older patients and transplant candidates (34).

However, it is important to recognize that most prognostic models were derived in Japanese populations treated within defined therapeutic eras. Their applicability to Western cohorts, differing treatment algorithms, and contemporary immunotherapeutic strategies remains incompletely validated (29, 37, 38). As therapeutic paradigms evolve, static clinical indices may require integration with molecular and treatment-specific variables.

Risk stratification is particularly relevant in the context of allogeneic hematopoietic stem cell transplantation (allo-HSCT). While allo-HSCT remains the only modality consistently associated with durable long-term remission in aggressive ATLL, transplant-related mortality and severe graft-versus-host disease substantially influence net survival benefit (10, 37, 39). Thus, prognostic assessment must balance relapse risk against non-relapse mortality, underscoring the need for improved biologically informed selection strategies.

### 5.3 Survival outcomes by subtype

Survival outcomes vary markedly across clinical subtypes and treatment context. Acute and lymphomatous ATLL are associated with the poorest outcomes under conventional chemotherapy-based approaches (6, 29), whereas chronic and smoldering subtypes demonstrate longer survival trajectories but retain risk of transformation (5, 29).

Reported survival differs across geographic regions and treatment eras. Outcomes in Japanese cohorts—where antiviral strategies, intensive chemotherapy regimens, and transplant consolidation are more systematically applied—may not directly extrapolate to Western populations (9, 37, 38). Furthermore, clinicogenomic heterogeneity and treatment selection bias complicate cross-cohort comparisons.

Collectively, these data highlight that prognosis in ATLL is not determined solely by clinical subtype but by a multidimensional interplay of host factors, proviral burden, somatic mutations, and therapeutic exposure. Future risk models will likely require integration of molecular variables with dynamic treatment response metrics to meaningfully improve prognostic precision.

## 6 Current treatment approaches

Therapeutic decision-making in adult T-cell leukemia/lymphoma (ATLL) is subtype-dependent and constrained by intrinsic chemoresistance, profound immunosuppression, and the limited durability of cytotoxic regimens. Importantly, most prospective therapeutic data derive from Japanese cohorts, where disease epidemiology, access to transplantation, and supportive care infrastructure differ from Western practice. Therefore, extrapolation to North American populations must be performed cautiously. A summary of NCCN-aligned first-line treatment approaches for ATLL in North America is provided in **Table 3**.

**Table 3:** NCCN-aligned first-line management of adult T-cell leukemia/lymphoma (North America).

ATLL subtype	Clinical situation/risk	Primary therapeutic goal	NCCN-aligned preferred initial approach (North America)*	Role of allo-HSCT	Key considerations
Acute type	Fit, transplant-eligible; leukemic and/or systemic disease	Rapid disease control, potential cure	Multi-agent induction chemotherapy used for aggressive PTCL (e.g. CHO(E)P, dose-adjusted EPOCH, hyper-CVAD or similar) with early referral to a transplant center	Strongly consider allo-HSCT in first CR/PR for eligible patients with donor available	Clinical trial preferred whenever possible; CNS evaluation/prophylaxis per risk; antiviral therapy (AZT/IFN-α) may be integrated in leukemic presentations according to institutional practice
Acute type	Elderly, frail, or major comorbidities; not transplant-eligible	Disease control and symptom relief	Attenuated CHOP-like or EPOCH-like regimens; AZT/IFN-α–based therapy as a less intensive option in leukemic forms; Mogamulizumab plus CHOP (Moga-CHOP) as a NCCN-listed option for transplant-ineligible patients	Generally not a candidate	Emphasis on symptom control, infection prophylaxis, and early palliative care involvement; treatment intensity individualized to performance status
Lymphoma type	Fit, transplant-eligible; nodal/extranodal bulky disease, minimal leukemic component	Disease control, potential cure	Aggressive PTCL-type induction (e.g. CHO(E)P, dose-adjusted EPOCH, hyper-CVAD or similar); for CD30^+^ disease, CH(P) plus brentuximab vedotin is a guideline-supported option	Allo-HSCT in first CR/PR is recommended/strongly considered for eligible patients	Management parallels other aggressive PTCL; assess for occult leukemic component and HTLV-1–related complications; clinical trial enrollment encouraged
Lymphoma type	Transplant-ineligible (age, comorbidities, frailty)	Disease control, palliation	CHOP-like or EPOCH-like chemotherapy; CH(P)+brentuximab vedotin for CD30^+^ disease when feasible	Usually not pursued	Dose-reduced regimens or single-agent approaches may be appropriate; integrate local radiotherapy for symptomatic sites
Chronic type – favorable risk	Low tumor burden, preserved counts, favorable ATL-PI/labs	Delay progression, maintain quality of life	AZT + IFN-α as preferred systemic therapy in leukemic, non-bulky disease; observation acceptable in truly asymptomatic patients	Consider only in selected younger patients with high-risk biology or early progression	Upfront CHOP-like chemotherapy is generally avoided; close monitoring for evolution to unfavorable chronic or acute-like phenotype
Chronic type – unfavorable risk	Leukemic disease with adverse features (e.g. high LDH, high β_2_-microglobulin, high tumor burden)	Disease control, potential cure in fit patients	Combination chemotherapy similar to acute/lymphoma type, often sequenced or combined with AZT/IFN-α in leukemic presentations	Allo-HSCT considered for fit patients who achieve CR/PR	Risk stratification tools (e.g. ATL-PI) guide intensity and transplant planning; early transplant referral recommended
Smoldering type	Limited skin and/or blood involvement, minimal symptoms	Prevent progression, preserve quality of life	Observation, skin-directed therapy and/or local RT for isolated lesions; AZT + IFN-α in leukemic smoldering disease	Rarely indicated; reserved for biologically high-risk or transforming cases	Conservative management is appropriate for classic smoldering ATLL; systemic chemo generally deferred unless clear progression or transformation

Exact regimen categories (e.g., Category 1 vs 2A) vary by NCCN version; clinicians should confirm current NCCN T-Cell Lymphoma Guidelines at time of use.

Summary of guideline-aligned initial treatment strategies for ATLL in North America, synthesizing NCCN T-Cell Lymphoma recommendations and expert consensus. Table structured by clinical subtype, therapeutic intent, and transplant eligibility.

ATLL, adult T-cell leukemia/lymphoma; PTCL, peripheral T-cell lymphoma; CR, complete remission; PR, partial remission; allo-HSCT, allogeneic hematopoietic stem cell transplantation; AZT, zidovudine; IFN-α, interferon-α; LDH, lactate dehydrogenase; RT, radiotherapy; ATL-PI, Adult T-cell Leukemia Prognostic Index.

Adapted and synthesized from NCCN Clinical Practice Guidelines in Oncology (NCCN Guidelines®): T-Cell Lymphomas (Adult T-Cell Leukemia/Lymphoma module), Version 1.2024 (40). Subtype definitions based on Shimoyama criteria (6) and WHO 5th edition classification (41). Survival and prevalence estimates are approximate and cohort-dependent.

### 6.1 Intensive chemotherapy for aggressive ATLL

For acute and lymphomatous subtypes, multi-agent chemotherapy remains the initial standard approach. In North America, dose-adjusted EPOCH is commonly used as a preferred cytotoxic backbone for aggressive ATLL, whereas mLSG15 (VCAP-AMP-VECP) is primarily used in Japan.

Historically, CHOP-based therapy demonstrated poor outcomes. In the Japan Clinical Oncology Group (JCOG) trials, conventional CHOP yielded complete response (CR) rates below 20–30% and median overall survival (OS) typically under 12 months in aggressive disease (7).

Dose-adjusted EPOCH has also been reported as a safe and effective treatment option for ATLL and is often favored in North American practice as a frontline backbone (42).

The JCOG9801 randomized phase III trial directly compared biweekly CHOP with the dose-intensified mLSG15 regimen (VCAP-AMP-VECP) (7). mLSG15 achieved:

- Higher complete response rates (40% vs 25%).
- Improved 2-year OS (31% vs 16%).
- Increased hematologic toxicity and infectious complications.

Although mLSG15 demonstrated statistically significant survival improvement, long-term outcomes remained poor, and treatment-related toxicity limited broad applicability. Furthermore, this study was conducted exclusively in Japanese centers, where earlier diagnosis and transplant referral patterns may influence survival estimates.

Thus, while mLSG15 represents the most rigorously studied cytotoxic platform, it does not provide durable disease control in the majority of patients and is best viewed as induction therapy rather than curative treatment. Initial therapy options for ATLL are summarized in **Table 4**.

**Table 4:** Initial therapy options for adult T-cell leukemia/lymphoma.

INITIAL THERAPY
Preferred (alphabetical order)
• Clinical trial • CHP (Cyclophosphamide/Doxorubicin/Prednisone) + Brentuximab vedotin for CD30+ cases • CHOP (Cyclophosphamide/Doxorubicin/Vincristine/Prednisone) + Mogamulizumab-kpkc^d,e^ (no intention to proceed to transplant) • Dose-adjusted EPOCH (Etoposide/Prednisone/Vincristine/Cyclophosphamide/Doxorubicin) • Interferon/Zidovudine^f^ (acute, chronic, and symptomatic smoldering subtypes)
Other Recommended (alphabetical order)
•CHOEP (Cyclophosphamide/Doxorubicin/Vincristine/Etoposide/Prednisone) • HyperCVAD (Cyclophosphamide/Vincristine/Doxorubicin/Dexamethasone) alternating with High-Dose Methotrexate/Cytarabine
Useful in Certain Circumstances
•CHOP (Cyclophosphamide/Doxorubicin/Vincristine/Prednisone) (unable to tolerate intensive regimen or non-CD30 expressing ATLL)

ATLL, adult T-cell leukemia/lymphoma; CHOP, cyclophosphamide/doxorubicin/vincristine/prednisone; CHOEP, cyclophosphamide/doxorubicin/vincristine/etoposide/prednisone; EPOCH, etoposide/prednisone/vincristine/cyclophosphamide/doxorubicin; hyper-CVAD, hyperfractionated cyclophosphamide/vincristine/doxorubicin/dexamethasone; AZT, zidovudine; IFN-α, interferon-α; allo-HSCT, allogeneic hematopoietic stem cell transplantation; PTCL, peripheral T-cell lymphoma.

### 6.2 Antiviral therapy (AZT + IFN-α)

In contrast to aggressive subtypes, chronic and smoldering ATLL demonstrate sensitivity to antiviral therapy.

A landmark meta-analysis by Hodson et al. demonstrated that zidovudine (AZT) plus interferon-α significantly improved survival in leukemic subtypes, particularly when administered as first-line therapy (9). In leukemic (acute and chronic) ATLL, antiviral therapy produced:

- 5-year OS ~46% in selected cohorts.
- Particularly favorable outcomes in patients without TP53 disruption.

Mechanistically, antiviral therapy appears most effective in cases with intact p53 function and lower genomic complexity, suggesting a biologically distinct subset responsive to proviral-targeting approaches.

However, antiviral therapy is insufficient for lymphomatous ATLL and high-burden aggressive disease. Additionally, most survival data derive from retrospective pooled analyses and non-randomized comparisons, limiting definitive causal inference.

Overall, AZT + IFN-α remains the preferred first-line backbone for chronic/smoldering and selected leukemic presentations, while lymphoma-type disease generally requires cytotoxic induction and/or antibody-based strategies. Because much of the survival signal derives from pooled retrospective cohorts, generalizability to contemporary non-Japanese practice and to patients treated with modern transplant or antibody sequencing remains an important limitation.

### 6.3 Mogamulizumab (anti-CCR4)

CCR4 is mutated or overexpressed in a substantial proportion of ATLL cases (27, 43), providing a rational therapeutic target.

In a phase II trial of relapsed ATLL, mogamulizumab produced an overall response rate (ORR) of 50%, with responses observed in both blood and nodal compartments (8). A randomized phase II study combining mogamulizumab with chemotherapy in newly diagnosed aggressive ATLL demonstrated improved CR rates compared to chemotherapy alone (44).

However, pre-transplant exposure to mogamulizumab has been associated with increased severe graft-versus-host disease (GVHD) and non-relapse mortality (NRM), likely due to depletion of regulatory T cells (45). However, transplant practice has evolved, and contemporary GVHD prophylaxis platforms—particularly post-transplant cyclophosphamide (PTCy)–based approaches—along with improved GVHD therapies (including ruxolitinib for steroid-refractory GVHD) may mitigate transplant-related mortality compared with earlier series (46–48). In addition, emerging data suggest the net impact of pre-allo mogamulizumab exposure may be context-dependent and may not uniformly worsen overall survival, underscoring the importance of patient selection, timing/washout, and prophylaxis strategy when integrating anti-CCR4 therapy into curative-intent pathways (49). Thus, while effective in disease control, mogamulizumab introduces transplant-specific risk tradeoffs that must be explicitly considered.

Importantly, most prospective mogamulizumab trials were conducted in Japan. Real-world outcomes outside endemic regions remain less well characterized (50).

### 6.4 Allogeneic hematopoietic stem cell transplantation

Allo-HSCT remains the only potentially curative approach for patients with aggressive ATLL.

Large Japanese registry analyses have demonstrated:

- 3-year OS ~36% following allo-HSCT (10).
- Improved outcomes when performed in first complete remission.

However, treatment-related mortality remains substantial. Nationwide analyses report non-relapse mortality rates exceeding 30%, particularly in older patients and those receiving prior mogamulizumab (45, 51). At the same time, modern GVHD prophylaxis (including PTCy-based strategies) and improved GVHD treatment options may be contributing to lower transplant-related mortality in more contemporary practice compared with historical cohorts (46–48).

Reduced-intensity conditioning regimens have expanded eligibility to older populations (52), but outcomes remain inferior compared with younger transplant recipients.

Thus, allo-HSCT represents a high-risk, high-reward intervention whose benefit is highly dependent on timing, remission status, and donor selection.

### 6.5 Limitations of current paradigms

Despite incremental improvements with intensified chemotherapy, antiviral therapy, monoclonal antibodies, and transplantation, overall survival for aggressive ATLL remains poor.

Key limitations include:

- Intrinsic genomic complexity (e.g., TP53, NF-κB pathway alterations) (17).
- Chemoresistance.
- Immunosuppression-related infectious mortality.
- Geographic concentration of trial data in Japan.

These factors underscore the need for biomarker-driven therapeutic stratification and rational integration of targeted and immune-based strategies. A summary of current treatment strategies by disease subtype is provided in **Table 5**.

**Table 5:** Treatment strategy by ATLL subtype.

ATLL subtype	Preferred initial strategy	Evidence base	Key considerations/risk tradeoffs
Acute	Intensive chemotherapy (dose-adjusted EPOCH favored in North America; mLSG15 mainly in Japan; preferred over CHOP) → allo-HSCT in CR1 if eligible	JCOG9801 randomized trial (7); transplant registry data (10)	Short median OS with chemotherapy alone; transplant offers potential long-term survival but high NRM; outcomes derived primarily from Japanese cohorts
Lymphomatous	Intensive chemotherapy (mLSG15 or CHOP) ± mogamulizumab → consider allo-HSCT in CR1	JCOG9801 (7); Mogamulizumab phase II (8, 44)	AZT/IFN ineffective; transplant timing critical; pre-transplant mogamulizumab ↑ severe GVHD risk (45)
Chronic (favorable)	AZT + IFN-α (first-line)	Meta-analysis and pooled cohort data (9)	Durable responses in leukemic subtype; best outcomes in TP53-intact disease; requires adherence and monitoring
Chronic (unfavorable)	AZT + IFN-α vs chemotherapy depending on tumor burden and LDH	Hodson meta-analysis (9); retrospective cohort data	Higher progression risk; consider early transplant evaluation in fit patients
Smoldering	Observation or AZT + IFN-α	Observational cohort data (9)	Risk of progression; requires longitudinal proviral load monitoring (5)

ATLL, adult T-cell leukemia/lymphoma; EPOCH, etoposide/prednisone/vincristine/cyclophosphamide/doxorubicin; mLSG15, VCAP-AMP-VECP regimen; allo-HSCT, allogeneic hematopoietic stem cell transplantation; CR1, first complete remission; OS, overall survival; NRM, non-relapse mortality; GVHD, graft-versus-host disease; AZT, zidovudine; IFN-α, interferon-α; LDH, lactate dehydrogenase. Synthesized from JCOG9801 and related chemotherapy studies (7), antiviral pooled analyses (9), mogamulizumab studies (12, 44, 45), and allo-HSCT registry data (17). Outcomes and tradeoffs are approximate and cohort-dependent.

#### 6.5.1 Treatment strategy by subtype

## 7 Novel therapeutic strategies

Adult T-cell leukemia/lymphoma (ATLL) remains therapeutically challenging. In aggressive subtypes, CHOP-based chemotherapy produces limited durability, and dose-intensified regimens such as VCAP-AMP-VECP (mLSG15) improve response rates but do not consistently achieve long-term disease control (7). Integrated genomic analyses demonstrating recurrent alterations in CCR4, TCR/NF-κB, JAK/STAT, and epigenetic regulators have provided a biologic framework for molecularly informed therapeutic strategies (15, 16).

For eligible patients achieving remission, allogeneic hematopoietic stem cell transplantation (allo-HSCT) remains the only intervention with established curative potential, although transplant-related mortality and GVHD significantly limit applicability (11, 37).

Notably, most prospective therapeutic datasets—particularly for intensified chemotherapy platforms, mogamulizumab combinations, and transplant sequencing—derive from Japan; therefore, we highlight where extrapolation to North American practice is based on limited real-world validation. Key NCCN-aligned options and novel agents for relapsed/refractory ATLL are summarized in **Table 6**.

**Table 6:** NCCN-aligned options and novel agents for relapsed/refractory adult T-cell leukemia/lymphoma.

Agent/strategy	Mechanism/class	Typical line/setting	Role in ATLL (North America)	Key caveats and practical points
Clinical trial	Varies (targeted, cellular, combination strategies)	Any relapse or refractory disease; also considered in frontline	Preferred option whenever available for R/R ATLL, given poor outcomes with standard therapies	Access to novel agents (e.g. epigenetic drugs, cellular therapies, combinations with antiviral or immunomodulatory backbones); recommended as first consideration in guidelines and consensus statements
Mogamulizumab	Defucosylated anti-CCR4 IgG1 mAb	Relapsed/refractory CCR4^+^ ATLL	Guideline-listed systemic option with meaningful single-agent activity in R/R disease; used as salvage or as a bridge to deeper cytoreduction	High risk of severe, steroid-refractory GVHD if allo-HSCT is performed soon after exposure; transplant teams generally require a washout period and careful GVHD prophylaxis
Lenalidomide	Oral immunomodulatory agent (IMiD)	Relapsed/refractory aggressive ATLL after prior systemic therapy	Oral option with documented activity in R/R ATLL; can provide disease control and symptom improvement in selected patients	Cytopenias, rash and infections are common; often used in patients not suitable for intensive chemo or as maintenance-type therapy in responders
Brentuximab vedotin (for CD30^+^ disease)	Anti-CD30 antibody–drug conjugate	R/R CD30^+^ ATLL; also incorporated in frontline regimens for CD30^+^ PTCL in some settings	For CD30-expressing ATLL, NCCN supports brentuximab-based regimens by extrapolation from PTCL data; used alone or combined with CH(P)-like backbones	Requires CD30 expression; neuropathy and cytopenias are dose-limiting; experience in ATLL is more limited than in other PTCLs but supported by consensus reports
Pralatrexate	Antifolate chemotherapy	R/R PTCL, including ATLL	Listed among active agents for relapsed PTCL; may be used in R/R ATLL when clinical trials or targeted options are unavailable	Mucositis and cytopenias frequent; folate and B12 supplementation and strict supportive care are required
HDAC inhibitors (romidepsin, belinostat)	Histone deacetylase inhibitors	R/R PTCL, occasionally used in ATLL	Can induce responses in R/R PTCL and are used in some ATLL cases when other options are limited	QT prolongation, cytopenias and infections require monitoring; growing interest in combining with antiviral or epigenetic strategies in mechanistically-driven trials
Conventional multi-agent salvage chemotherapy (e.g. ICE, GDP, DHAP, ESHAP)	Cytotoxic chemotherapy combinations	R/R ATLL with transplant-eligible patient and chemosensitive disease	Used to debulk disease and achieve remission as a bridge to allo-HSCT in fit patients	Responses are often short-lived; toxicity substantial in heavily pretreated or frail patients; should be coupled with rapid transplant planning
Allo-HSCT in relapse	Graft-versus-tumor effect from donor immune system	Chemo-sensitive relapse in fit patient with donor	Only modality with curative potential in R/R ATLL; considered in patients who achieve at least PR with salvage therapy	High non-relapse mortality and relapse risk; outcomes best when disease burden is low; prior mogamulizumab exposure increases GVHD risk and must be factored into transplant planning
Palliative/supportive care	Symptom-directed measures (steroids, RT, transfusions, infection prophylaxis, pain control)	Advanced, refractory disease or patients not candidates for additional systemic therapy	Central component of care across all stages; becomes primary focus in multiply relapsed, refractory, or frail patients	Clear goals-of-care discussions, integration of palliative care, and early hospice referral are recommended to optimize quality of life

Therapies and sequencing should be aligned with the most recent NCCN T-Cell Lymphoma Guidelines (Adult T-Cell Leukemia/Lymphoma module).

R/R, relapsed/refractory; allo-HSCT, allogeneic hematopoietic stem cell transplantation; GVHD, graft-versus-host disease; IMiD, immunomodulatory drug; PTCL, peripheral T-cell lymphoma.

Therapeutic options aligned with NCCN Clinical Practice Guidelines in Oncology: T-Cell Lymphomas, Version 1.2024 (40). Recommendations should be interpreted in the context of patient fitness, transplant eligibility, and evolving evidence.

Treatment strategies for relapsed/refractory ATLL consistent with NCCN T-Cell Lymphoma guidance. Emphasizes approved agents, transplant pathways, and mechanisms relevant to clinical decision-making in North America.

### 7.1 Anti-CCR4 therapy: mogamulizumab

CCR4 is highly expressed in ATLL and frequently mutated (27). Mogamulizumab (KW-0761), a defucosylated anti-CCR4 monoclonal antibody, demonstrated clinical activity in relapsed/refractory ATLL in a multicenter phase II study, establishing it as an active single agent in this population (8).

In a randomized phase II study in newly diagnosed aggressive ATLL, addition of mogamulizumab to chemotherapy improved response endpoints compared with chemotherapy alone (44). However, long-term survival impact remains dependent on disease biology and consolidation strategies.

Because these pivotal studies were Japan-based, the magnitude of benefit and toxicity trade-offs may differ in non-endemic regions with different baseline comorbidities, HTLV-1 epidemiology, and transplant practice patterns.

Genomic data demonstrate gain-of-function CCR4 mutations in a substantial subset of ATLL cases (27). Correlative analyses have suggested improved outcomes with mogamulizumab in patients harboring CCR4 mutations (43), supporting a biologically plausible predictive relationship, although prospective biomarker-driven validation remains lacking.

A critical limitation involves transplant sequencing. Pre-transplant exposure to mogamulizumab has been associated with increased severe GVHD and non-relapse mortality (45). This observation has influenced clinical practice toward avoiding close pre-transplant administration when allo-HSCT is planned.

### 7.2 Targeting TCR/NF-κB and JAK/STAT signaling

Comprehensive genomic profiling demonstrates convergence of somatic alterations on TCR/NF-κB and JAK/STAT pathways (15, 16). Mechanistically, loss of miR-31 has been shown to promote NIK-dependent NF-κB activation in ATLL cells (24), while Tax-driven signaling stabilizes anti-apoptotic programs such as MCL-1 through NF-κB activation (12).

These findings provide strong mechanistic rationale for pathway-directed therapies. However, clinical translation remains early, and robust phase II or III pathway-specific inhibitor data in ATLL are limited. At present, these approaches remain biologically rational but not yet practice-defining.

### 7.3 Allogeneic hematopoietic stem cell transplantation

Registry data from Japanese cohorts demonstrate that allo-HSCT can produce durable survival in a subset of patients with aggressive ATLL (10, 37). EBMT analyses similarly confirm curative potential in selected patients while emphasizing high non-relapse mortality (11).

Evidence for graft-versus-leukemia activity is supported by immune responses against HTLV-1 antigens after transplant (53), suggesting immunologic disease control beyond cytotoxic conditioning. However, GVHD remains a major contributor to morbidity and mortality (51).

Outcomes are inferior when transplant is performed in relapsed/refractory disease (54), reinforcing the importance of remission status and early referral in transplant-eligible patients.

### 7.4 Antiviral therapy: zidovudine + interferon-α (± Arsenic)

Antiviral therapy represents a biologically distinct strategy targeting HTLV-1–driven leukemogenesis. Observational analyses have demonstrated improved survival in patients receiving AZT/IFN-α–based therapy, particularly in leukemic presentations (9).

A phase II study evaluating arsenic trioxide combined with IFN-α and AZT demonstrated activity in chronic ATLL, supporting a viral-directed differentiation strategy in select settings (55). These data provide proof-of-concept for therapies that exploit viral dependence rather than purely cytotoxic mechanisms.

### 7.5 Apoptosis and cell-cycle vulnerabilities

ATLL frequently exhibits TP53 alterations associated with adverse outcomes (35, 36). Mechanistically, Tax-mediated NF-κB signaling enhances anti-apoptotic signaling including MCL-1 stabilization (12), contributing to chemotherapy resistance.

These observations highlight apoptosis and tumor suppressor dysfunction as key resistance axes, although targeted therapies exploiting these vulnerabilities remain investigational.

### 7.6 Immunomodulatory drugs: lenalidomide

Lenalidomide has demonstrated activity in relapsed/refractory ATLL. A phase I study established feasibility and early signals of response (56), followed by a multicenter phase II trial reporting objective responses in heavily pretreated patients (57). Additional prospective data support its use as a salvage option (58). Beyond salvage therapy, lenalidomide has also been explored as a maintenance strategy in ATLL, with emerging clinical experience suggesting a potential role in prolonging disease control in selected patients (59).

While response rates are modest, lenalidomide offers a non-cytotoxic strategy with manageable toxicity in selected patients.

### 7.7 Immune checkpoint inhibitors: cautionary experience

Unlike many hematologic malignancies, PD-1 blockade has been associated with rapid disease acceleration in ATLL. A clinical report described fulminant progression following PD-1 inhibitor therapy (18), raising substantial safety concerns. Consequently, immune checkpoint inhibition is not recommended outside carefully controlled clinical trials.

### 7.8 Emerging epigenetic strategies

Epigenetic dysregulation is a recurrent feature of ATLL biology (15, 26). Valemetostat, a dual EZH1/2 inhibitor, demonstrated activity in a phase II study in relapsed/refractory ATLL (60), representing one of the first epigenetic therapies with prospective clinical data in this disease.

These results illustrate how genomic insights may translate into mechanism-based therapies, although longer follow-up and comparative data are required.

### 7.9 An integrative therapeutic model for ATLL

ATLL pathogenesis reflects a multistep convergence of (i) persistent HTLV-1–driven proliferative signaling (Tax/HBZ), (ii) acquisition of somatic alterations activating TCR/NF-κB and JAK/STAT pathways, (iii) immune-evasion mechanisms including CCR4 overexpression and checkpoint modulation, and (iv) progressive genomic instability culminating in chemotherapy resistance (12, 15, 16, 24, 27). From a therapeutic standpoint, these layers can be conceptualized as three interdependent axes: viral dependence, oncogenic pathway addiction, and immune context vulnerability. Antiviral therapy (AZT/IFN-α ± arsenic) targets the viral dependence axis (9, 55); mogamulizumab exploits immune context vulnerability via CCR4-directed cytotoxicity (8, 43); and allo-HSCT leverages graft-versus-leukemia immunity to overcome intrinsic chemoresistance (37, 53). Emerging epigenetic therapies such as dual EZH1/2 inhibition further suggest that chromatin remodeling may represent a fourth actionable layer (26, 60). Framing ATLL management through this integrative model highlights the need for rational sequencing and biologically informed combination strategies rather than reliance on intensification of cytotoxic chemotherapy alone.

## 8 Challenges and future directions

Whereas Section 7 summarizes therapies supported by primary mechanistic or clinical evidence in ATLL, this section focuses on the key translational barriers and research agenda required to implement biology-adapted care (biomarker validation, sequencing, transplant integration, and prevention).

Despite improvements in molecular characterization and targeted therapy development, overall survival for aggressive ATLL remains poor, even with dose-intensified chemotherapy and transplant consolidation (7, 11, 37). Integrated genomic analyses demonstrate marked interpatient heterogeneity and convergence on recurrent pathway-level vulnerabilities, supporting a precision-guided therapeutic framework rather than uniform treatment approaches (15–17). Future progress will depend on translating these molecular insights into rationally sequenced, biology-adapted strategies.

### 8.1 Molecular stratification and predictive biomarkers

Comprehensive sequencing studies have defined recurrent alterations affecting CCR4, TCR/NF-κB signaling, epigenetic regulators, and tumor suppressor pathways (15–17). Gain-of-function CCR4 mutations were identified in a substantial subset of cases (27), and clinical correlative analyses demonstrated improved responses to mogamulizumab in CCR4-mutated disease (43). These findings support CCR4 mutation status as a candidate predictive biomarker for anti-CCR4 therapy.

Similarly, TP53 alterations have been associated with inferior outcomes (35, 36), suggesting potential utility for risk-adapted intensification or investigational strategies in high-risk molecular subsets. Future clinical trial design in ATLL should incorporate prospective molecular stratification based on these recurrent genomic lesions rather than treating ATLL as a biologically homogeneous entity.

### 8.2 Targeting NF-κB and epigenetic dependencies

Mechanistic studies demonstrate that HTLV-1–driven oncogenesis converges on persistent NF-κB activation, including miR-31 loss leading to NIK-dependent NF-κB signaling (24), and Tax-mediated stabilization of anti-apoptotic programs such as MCL-1 (12). These data provide a biological rationale for pathway-directed inhibition of NF-κB and downstream survival signals, although clinical validation remains limited.

Epigenetic dysregulation is also central to ATLL biology. Polycomb-dependent chromatin remodeling has been demonstrated in primary ATLL samples (26), and clinical evaluation of the dual EZH1/2 inhibitor valemetostat in relapsed/refractory ATLL showed objective responses in a phase II study (60). These findings support epigenetic targeting as an evidence-supported therapeutic axis rather than a purely theoretical approach.

Despite strong pathway rationale, single-agent clinical activity of JAK/STAT and NF-κB-directed approaches has been modest, likely reflecting pathway redundancy, concurrent anti-apoptotic programs, subclonal heterogeneity, and the challenge of achieving therapeutic inhibition in a disease marked by profound immune dysregulation and treatment-limiting infections.

### 8.3 Refining antiviral and viral-directed strategies

Given the viral etiology of ATLL, antiviral therapy remains biologically rational. Observational analyses demonstrated improved outcomes with AZT/IFN-α–based strategies in leukemic presentations (9), and a phase II study combining arsenic, interferon-α, and AZT showed activity in chronic ATLL (55). These clinical data support continued refinement of viral-directed strategies, particularly in indolent or leukemic subtypes.

At a mechanistic level, proviral load and clonality correlate with disease progression risk (4, 5, 14), suggesting potential utility of molecular viral monitoring in treatment adaptation and risk stratification. Prospective validation of proviral dynamics as a response biomarker represents an important research direction.

### 8.4 Immune modulation: lessons from checkpoint inhibition

Unlike many malignancies, PD-1 blockade has been associated with rapid disease acceleration in ATLL (18). This paradoxical outcome underscores the unique immune biology of HTLV-1–driven disease and highlights the necessity of mechanistic grounding before applying immunotherapy paradigms extrapolated from other cancers. Future immune-based strategies must be guided by a detailed understanding of viral-host immune interactions rather than empirical extrapolation.

While the most compelling clinical signal is in ATLL, the observation raises broader caution for PD-1 blockade in virus-driven T-cell neoplasms in which malignant cells may retain a PD-1–dependent restraint phenotype; however, evidence outside ATLL remains limited, and generalizability is currently speculative.

### 8.5 Optimizing transplant integration

Allo-HSCT remains the only intervention with established curative potential for selected patients (10, 11, 37). Registry studies demonstrate long-term survival in a subset, particularly when performed in remission (37, 39), but outcomes are limited by substantial non-relapse mortality and GVHD (11, 51). Pretransplant exposure to mogamulizumab has been associated with increased severe GVHD and non-relapse mortality (45), emphasizing the importance of treatment sequencing and washout planning.

Risk-mitigation strategies used in practice include avoiding mogamulizumab when imminent allo-HSCT is planned, enforcing an adequate washout interval prior to conditioning, and intensifying GVHD prophylaxis and monitoring given the drug’s depletion of CCR4^+^ regulatory T cells.

Future strategies should focus on improving pretransplant disease control through molecularly guided induction and optimizing post-transplant immune balance, preserving graft-versus-leukemia effects while limiting toxicity.

### 8.6 Global HTLV-1 prevention

ATLL incidence reflects the epidemiology of HTLV-1 infection (3). Mother-to-child transmission remains a major route of viral spread in endemic regions (20). Long-term reduction in ATLL burden will require sustained public health interventions targeting HTLV-1 screening and transmission prevention. Integration of epidemiologic data with clinical and molecular advances is essential for comprehensive disease control.

Taken together, the therapeutic advances outlined in Section 7 and the forward-looking strategies discussed in this section support an integrated disease model in which ATLL is driven by persistent viral oncogenesis layered upon recurrent host genomic alterations that converge on NF-κB signaling, immune evasion, and epigenetic reprogramming (15–17, 24, 26). In this framework, antiviral therapy, CCR4-directed immunotherapy, epigenetic targeting, and transplant consolidation are not independent modalities but biologically complementary interventions that address distinct nodes within a shared pathogenic network. Future progress in ATLL will therefore depend less on incremental addition of single agents and more on rational sequencing and molecularly informed combination strategies that align treatment intensity with subtype biology, mutational profile, and transplant eligibility. By explicitly linking genomic architecture to therapeutic decision-making, this integrative model provides a translational scaffold that may help refine risk-adapted care and differentiate this review from purely descriptive summaries of existing literature.

## 9 Conclusion

Adult T-cell leukemia/lymphoma (ATLL) remains one of the most biologically complex and therapeutically refractory mature T-cell neoplasms. Its pathogenesis reflects a multistep process initiated by HTLV-1–driven proliferative signaling (Tax/HBZ-mediated NF-κB activation) and sustained by the progressive accumulation of somatic alterations in TCR/NF-κB, JAK/STAT, epigenetic, and immune-escape pathways, as defined by integrated genomic analyses (15, 16, 24, 27). Clinically, outcomes remain poor for aggressive subtypes despite dose-intensified chemotherapy regimens such as VCAP-AMP-VECP (7), and durable disease control is achieved primarily in selected patients undergoing allogeneic hematopoietic stem cell transplantation, where graft-versus-leukemia effects must be balanced against substantial non-relapse mortality (11, 37, 51).

Therapeutic advances, including CCR4-directed therapy with mogamulizumab (8, 27, 43, 44), antiviral strategies with AZT/interferon-α (9, 55), and immunomodulatory agents such as lenalidomide (56, 57), illustrate the transition from empiric cytotoxic therapy toward mechanism-informed treatment. However, clinical heterogeneity, transplant-related toxicity, and the risk of immune dysregulation—particularly in the context of checkpoint inhibition (18)—underscore the need for biologically integrated treatment strategies.

As outlined in the Integrative Model proposed in this review, future progress in ATLL will depend on rational sequencing of viral-directed therapy, pathway-targeted agents, and transplant-based consolidation, guided by genomic risk features and biomarker-driven patient selection. Parallel investment in HTLV-1 prevention and early detection remains essential to altering the global disease burden (3, 20). Bridging mechanistic insight with clinically validated, biomarker-informed intervention strategies represents the most promising path toward improving long-term outcomes in this rare but devastating malignancy.
